# Surface Cleaning with a Microfiber Cloth and Water followed by Ultraviolet-C Light Exposure Achieves Non-Inferior Disinfection of a Pathogenic Staphylococcus aureus Strain versus Use of Germicidal Wipes.

**DOI:** 10.7759/cureus.65963

**Published:** 2024-08-01

**Authors:** Stephanie Gibbons, Franklin Dexter, Randy W Loftus, Brendan T Wanta, Carmen T Brindeiro, Soyun M Hwang, Jonathan E Charnin

**Affiliations:** 1 Anesthesia, University of Iowa, Iowa City, USA; 2 Anesthesiology and Perioperative Medicine, Mayo Clinic, Rochester, USA; 3 Microbiology, RDB Bioinformatics, Coralville, USA; 4 Anesthesiology, Mayo Clinic, Rochester, USA

**Keywords:** uv-c, ultraviolet-c, surface disinfection, sequence type 5, staphylococcus aureus

## Abstract

Background: We hypothesized that ultraviolet-C (UV-C) irradiation (Surfacide, Waukesha, WI) following use of microfiber cloths (Sanny Shop LLC, Longmont, CO) soaked in water would be noninferior to surface disinfection wipes containing a quaternary ammonium compound and alcohol (PDI Healthcare, Woodcliff Lake, NJ) for the pathogenic *Staphylococcus aureus* (*S. aureus*) sequence type 5 (ST5).

Methods: This was a randomized laboratory study of disinfection approaches for *S. aureus* ST5. A total of 270 polycarbonate slides loaded with ST5 were prepared for the standard surface disinfection group (N=18) and water-soaked microfiber cloths and UV-C treatment group (N=144), along with positive and negative microbiological controls.

Results: All 18 samples of *S. aureus* ST5 bacteria treated with standard chemical wipes showed complete disinfection (colony forming units (CFU) = 0). All 144 treatments with water-soaked microfiber wipes followed by UV-C exposure showed complete disinfection (CFU =0) regardless of soiling, height from the floor, or orientation to the emitters. The upper 95% exact one-sided confidence limit for any CFU >0 was 2.1%.

Discussion: These data affirm our hypothesis that surface wiping with a damp cloth followed by triangular UV-C irradiation delivery is noninferior to surface disinfection for *S. aureus* ST5 using germicidal wipes, even when UV-C is compromised by height from the floor and orientation to the emitters and surface disinfection is targeted.

Conclusion: Removing bioburden with chemical-free microfiber cloths followed by triangular UV-C delivery is a noninferior strategy to targeted surface disinfection with chemical disinfecting wipes for the pathogenic *S. aureus* ST5 strain in the laboratory setting.

## Introduction

Bacterial pathogens in the operating room that survive cleaning efforts can contribute to surgical site infection (SSI) and patient harm [1]. The anesthesia workspace is particularly prone to microbial contamination due to the high task density associated with anesthesia care and reducing surface contamination has been the target of infection mitigation strategies [2,3].

Contamination of the anesthesia machine is associated with increased bacterial burden. When the magnitude of contamination reaches a threshold of ≥100 colony forming units (CFU), there is increased risk of intravenous stopcock contamination, major bacterial pathogen detection, and SSI [1,4,5].

Surface cleaning can be effective at reducing bacterial contamination [6]. However, adequate disinfection is not performed in routine practice [4]. A recent study demonstrated that over 40% of measured operating room environmental sites returned over 100 CFU after terminal cleaning [4]. Causes for failure are understood. Although chemical disinfection has near complete efficacy [6], effectiveness depends on wiping all surfaces which is not practical [7]. Thus, further innovation for anesthesia work area cleaning is indicated.

Ultraviolet-C (UV-C) disinfection irradiates nearly the entire surface, has a mechanism of action that is distinct from that of chemical disinfectants [8], and is bactericidal and viricidal at sufficient doses [8,9]. Studies that have incorporated some component of UV-C irradiation to augment usual surface disinfection processes of the anesthesia workspace have shown a reduction in bacterial/viral transmission [10,11, 12] and reduced risk of SSI [13,14]. Further, a recent study showed that a triangular UV-C delivery system was effective against a highly pathogenic bacterium, *Staphylococcus aureus *(*S. aureus*)* *sequence type 5 (ST5), even with factors that limited dose delivery such as target orientation, distance, height, and delivery configuration [15].

One knowledge gap is whether soiling (e.g., saliva, mucus, blood, water, or colloid) reduces the efficacy of UV-C disinfection. Soiling is a relevant consideration for the operating room where there is frequent exposure to patient bodily fluids. Another knowledge gap is the relative effectiveness of removal of bioburden with a microfiber cloth vs. removal of the bioburden and treatment with a disinfecting chemical. This is an important consideration, as it may be that use of potentially harmful chemicals, for providers and the environment, can be reduced [16].

We hypothesized that use of a microfiber cloth (Sanny Shop, Longmont, CO) dampened with water to wipe surfaces prior to UV-C irradiation (Surfacide, Waukesha, WI) would address soiling, and thus, that UV-C would be a noninferior approach to targeted surface disinfection with a microfiber cloth soaked in quaternary ammonium and alcohol chemicals (PDI Healthcare, Woodcliff Lake, NJ), regardless of horizontal orientation to the emitters or increased height from the floor of the environmental target. Our secondary hypothesis was that the dose of UV-C delivered would be sufficient for bactericidal activity against a variety of pathogens [17]. We conducted a laboratory study using the more pathogenic *S. aureus *ST5 to test these hypotheses [15].

## Materials and methods

This was a randomized laboratory study conducted at RDB Bioinformatics (Coralville, IA, 52241) from November 21, 2023, to January 15, 2024. Our primary aim was to compare residual *S. aureus* ST5 CFU following two cleaning approaches: 1) pretreatment of contaminated surfaces with a microfiber cloth dampened with water followed by 21 minutes of triangular treatment with UV-C; and 2) use of a microfiber cloth soaked in a quaternary ammonium compound and alcohol. Our secondary aim was to quantify the dose of UV-C irradiation delivered to target sites at a variety of heights from the floor and orientation to the emitters over 21 minutes of triangular UV-C treatment. 

A total of 270 polycarbonate slides were prepared for the two study groups, UV-C and microfiber treatment group (N=144) and the surface disinfection group (N=18), along with positive and negative microbiological controls (Table 1).

**Table 1:** Slide Treatment Assignments UV-C: Ultraviolet-C

Treatment Group	Height (Inches)/Orientation	Lag Time (Hours)	Number of Slides
Chemical Wipe	Positive control		9
	Negative control		9
		0	9
		3	9
UV-C Not Soil	Positive control		18
	Negative control		18
	47.5/Vertical	0	9
		3	9
	69.5/Horizontal	0	9
		3	9
UV-C Soiled with FBS	Positive control		9
	Negative control		9
	47.5/Vertical	0	9
		3	9
	69.5/Horizontal	0	9
		3	9
UV-C Soiled with Artificial Saliva	Positive control		9
	Negative control		9
	47.5/Vertical	0	9
		3	9
	69.5/Horizontal	0	9
		3	9
UV-C Soiled with Sheep's Blood	Positive control		9
	Negative control		9
	47.5/Vertical	0	9
		3	9
	69.5/Horizontal	0	9
		3	9
Total			270

An *S. aureus* ST5 glycerol stock was randomly selected from the bacterial archive, streaked to a 5% sheep’s blood agar (SBA) plate (Thermo Fisher Scientific, Waltham, MA), and incubated for 24 hours at 36°C. *S. aureus *ST5 CFU were used to generate a 4.0 McFarland Standard with sterile, DNase/RNase-free distilled water (Thermo Fisher Scientific, Waltham, MA) to achieve a final volume of 1,900 µL. The soiling agent (100 µL of sterile, DNase/RNase-Free distilled water, fetal bovine serum (Thermo Fisher Scientific, Waltham, MA), sheep's blood (Thermo Fisher Scientific, Waltham, MA), or artificial saliva (Pickering Laboratories Inc, Mountain View, CA)) was added to the bacterial suspension to create a final volume of 2.0 mL. Then, the solution was vortexed for five seconds and ten 1 µL drops of the final solution placed onto polycarbonate slides (Thomas Scientific, Chadds Ford Township, PA). They were then allowed to dry under a laminar flow hood for six hours.

A total of 100 µL of sheep’s blood, artificial saliva, or fetal bovine serum was added to 1,900 µL of sterile, DNase/RNase-free water, the solution vortexed for five seconds, and ten 1 µL drops placed onto each slide and dried for six hours under a laminar flow hood.

Cleaning involved either use of a microfiber cloth dampened with sterile, DNase/RNase-free water to wipe a slide followed by 21 minutes of UV-C treatment via the triangular configuration or cleaning with a microfiber cloth soaked in a disinfectant. We chose 21 minutes of treatment because it is an intended use setting for operating room disinfection involving the UV-C emitters employed.

For cleaning with the cloth and UV-C, a microfiber cloth dampened with sterile, DNase/RNase-free water was pressed to a contaminated slide placed on the benchtop using a force similar to that needed to depress a keyboard key while wiping in one motion in a single direction. The slides were then affixed to an aluminum stand in orientations that were either vertical or horizontal to three UV-C emitters placed in a triangular configuration about the stand at 9 ft. The vertically oriented slides were positioned 47.5 inches from the floor, and the horizontally oriented slides were positioned 69.5 inches from the floor (Table 1). Radiometers (International Light Technologies, Inc, Peabody, MA) calibrated to 254-nm irradiation ±7.9, the peak intensity of UV-C, were positioned next to treatment slides to measure the band of wavelengths surrounding the peak intensity wavelength. Irradiance, W/cm^2^, or power/cm^2^ delivered to the carriers at a given distance was measured by the radiometers, where the dose delivered to the carriers was a function of time of irradiance exposure, W/cm^2^ X time (seconds) of exposure, or J/cm^2^. Each of the three UV-C emitters rotated to allow 360 degrees of motion. UV-C treatment was for 21 minutes. Per the manufacturer, the low-pressure lamps of the UV-C emitters employed were constructed with quartz envelopes that precluded emission of <240 nm wavelengths and, therefore, ozone generation.

Surface disinfection cleaning consisted of wiping the slide with a germicidal disposable wipe with bactericidal activity. Each slide was again wiped once on a lab bench with contact pressure like the force needed to depress a keyboard using one motion in a single direction.

Half of the slides were processed immediately and half three hours from the treatment. In the case of UV-C, the delay was to allow for the possibility of photoreactivation and dark repair. In the case of targeted surface disinfection cleaning, the delay was to allow for regrowth following disinfection and to account for manufacturer recommendations for cleaning time [18]. Slides were prepared in batches.

For each slide, a total of 10 mL of phosphate buffered saline (PBS) (Thermo Fisher Scientific, Waltham, MA) were added to a 50 mL conical tube (Thermo Fisher Scientific, Waltham, MA), and 990 µL and 900 µL of PBS were added to each of two 2 mL microcentrifuge tubes (Thermo Fisher Scientific, Waltham, MA). Each slide was placed into the 10 mL of PBS in the 50 mL conical tube and vortexed for 30 seconds. This solution was then serially diluted as follows: 10 µL of the 10 mL was added to the tube with 990 µL of PBS, vortexed for five seconds, and then 100 µL from that tube was added to the tube with 900 µL. Finally, 100 µL from each of the two 2 mL tubes were plated to 5% SBA and incubated over night at 36°C and CFU quantified.

This laboratory study was designed to determine whether UV-C treatment after use of microfiber cloths dampened with water to address soiling (treatment) was a noninferior approach to surface disinfection using chemicals (standard) within a non-inferiority margin of 4.5%. There would be 3:1 allocation between the UV-C group versus chemical (standard) group because of the three positions. Based on an expected percentage of 1.0% with residual CFU in both groups, then to achieve 80% power at a 0.05 alpha level would require 40 in the standard group and 121 in the UV-C groups [19,20].

After 18 samples had been processed with chemical treatment, we observed that there were 0 (0%) with any residual colonies despite measurable growth in our 54 positive control slides. In response, we stipulated that targeted chemical treatment was likely to be completely effective (i.e., CFU = 0). Therefore, no extra samples would receive chemical treatment because our goal was to test noninferiority of UV-C to optimized surface disinfection (targeted, a known site of contamination, distinct from clinical practice where the site of contamination is not necessarily known), and where additional surface disinfection treatment samples may weaken the standard (UV-C would then be compared to a less effective control). We planned that non-inferiority would be shown by the exact 95% upper one-sided confidence limit for CFU = 0 with UV-C being no greater than the non-inferiority margin of 4.5% [21].

## Results

Among the 54 positive control samples, all 54 recovered CFU (100%) (Table 2). 

**Table 2:** Positive Control Samples CFU: Colony forming units

Soiling Agent	Number of Samples	Number with Microbial Growth	Mean Number of CFU	Standard Deviation of CFU
Sterile Water	27	27	40	30
Fetal Bovine Serum	9	9	67	27
Artificial Saliva	9	9	43	17
5% Sheep's Blood	9	9	340	188

The highest sample mean CFU of recovered bacteria was from the group that involved soiling with 5% sheep’s blood. All cultures from negative control samples showed no growth.

All 144 treatments with water dampened microfiber wipes followed by UV-C showed complete disinfection (CFU = zero) regardless of soiling, height from the floor, or orientation to the emitters (Table 3).

**Table 3:** UV-C Treated Samples UV-C: Ultraviolet-C; CFU: Colony forming units

Soilage	Height (Inches)	Orientation	Hours before Treatment	Number of Slides	CFU > 0	% > 0
Artificial Saliva	47.5	Vertical	0	9	0	0%
Artificial Saliva	47.5	Vertical	3	9	0	0%
Artificial Saliva	69.5	Horizontal	0	9	0	0%
Artificial Saliva	69.5	Horizontal	3	9	0	0%
Blood	47.5	Vertical	0	9	0	0%
Blood	47.5	Vertical	3	9	0	0%
Blood	69.5	Horizontal	0	9	0	0%
Blood	69.5	Horizontal	3	9	0	0%
Fetal Bovine Serum	47.5	Vertical	0	9	0	0%
Fetal Bovine Serum	47.5	Vertical	3	9	0	0%
Fetal Bovine Serum	69.5	Horizontal	0	9	0	0%
Fetal Bovine Serum	69.5	Horizontal	3	9	0	0%
Water	47.5	Vertical	0	9	0	0%
Water	47.5	Vertical	3	9	0	0%
Water	69.5	Horizontal	0	9	0	0%
Water	69.5	Horizontal	3	9	0	0%

The upper 95% exact one-sided confidence limit for any CFU >0 would therefore be 2.1%. All 18 samples of *S. aureus* ST 5 bacteria treated with the standard chemical wipes also showed complete disinfection (CFU = 0).

The cumulative doses recorded for slides positioned vertically at 47.5 inches from the floor and slides positioned horizontally at 69.5 inches from the floor were comparable, mean (standard deviation) of 362.95 (11.58) and 360.97 (12.71) mJ/cm^2^, respectively.

## Discussion

Multiple reservoirs contribute to bacterial transmission among anesthesia workspace reservoirs. In turn, bacterial transmission is directly linked to SSI and other healthcare-associated infections [1,5,15,22]. A multifaceted improvement strategy has proven efficacy and effectiveness for generating substantial reductions in bacterial transmission and SSIs [10,13,14]. This includes improved frequency and quality of cleaning of the anesthesia machine, a potent transmission vehicle [22].

Prior work has investigated cleaning the surface of anesthesia machines and objects representative of machine knobs with water-soaked gauze and five different cleaning cloths [23]. However, cleaning efficacy was not defined by a clinically relevant bacterial reduction below 100 CFU [1,4]. This is an important gap, as environmental samples that return greater than or equal to 100 CFU are at increased risk for isolation of major bacterial pathogens [4]. Despite current cleaning processes, a significant proportion of measures reservoirs meet or exceed this 100 CFU threshold [7,24]. Moreover, there is evidence for resistance to chemical disinfectants [25]. Together, these findings provide impetus for improved and alternative disinfection approaches.

The minimal effective dose of UV-C irradiation for *S. aureus* ST 5, a pathogen that is frequently isolated from anesthesia work area reservoirs, was previously shown to be 1.88 ± 0.44 mJ/cm^2^ at horizontal orientation to the emitters [26]. Importantly, the dose could be delivered in as little as two minutes, translating to a practical recommendation for augmentation of surface disinfection of the anesthesia machine. These findings were particularly important for terminal cleaning procedures where failed cleaning may result in longitudinal transmission of more pathogenic, antibiotic resistant strain characteristics [15]. While factors such as orientation, distance, height, minimal effective dose to reduce risk of photo reactivation and dark repair, and delivery configuration were addressed, the study was limited by failure to address the potential impact of soiling and the relative efficacy of reducing the microbial bioburden.

In this study, we addressed the above limitations. We found that UV-C treatment following use of a microfiber cloth dampened with water was noninferior to targeted surface disinfection without soiling despite height from the floor and orientation to the emitters. There was no growth in either treatment group (zero CFU) for any study condition, thus achieving a clinically relevant reduction [1,4]. The 95% upper confidence interval for treatment failure was 2.1%. These study results demonstrate efficacy of UV-C (noninferiority <4.5%) when preceded by the removal of soil, showing potential for reduced use of chemicals for routine surface disinfection processes. To our knowledge, this is the first study to test this hypothesis. We also found that > 350mJ/cm2 was delivered regardless of orientation or height after a treatment period of 21-minutes. This is an important finding, as this dose will likely be effective for other vegetative, spore forming, and perhaps fungal pathogens that may be encountered in the operating room, the latter particularly important given the heightened role of environmental spread [9]. Further work is indicated to test the efficacy of this approach against these additional pathogens. Subsequently, a clinical trial should be designed to test efficacy and effectiveness vs. usual care, including consideration of variation in the disinfecting wipes employed, UV-C emitters, and/or bacterial strains. The clinically relevant endpoint for the trial should be <100 CFU of all cultured bacteria because among thousands of samples and >100 operating rooms, CFU <100 was associated with small risk of *S. aureus* isolation [4].

While the UV-C technology described in this study is non-ozone generating given use of low-pressure lamps with quartz envelopes that preclude emission of < 240 nm wavelengths of light, some UV-C technology produces ozone [27]. Thus, future study should quantify ozone generation during UV-C delivery with a variety of devices. Some devices can reach clinically relevant thresholds (0.1 ppm) within a one-hour exposure time [27]. Hence, measurements should be obtained at various times throughout the treatment period and after treatment to ensure that safe exposure levels below 0.1 ppm are maintained. Obviously, if irradiation cannot reach a site, such as the inside of a drawer, this would require surface disinfection, unless addressed by strategic positioning of equipment (e.g., opening the drawers before treatment). Future dosimetry study should be employed to test a variety of object positions in operating rooms. Changes in the UV-C delivery configuration and/or treatment time as described in this study may impact the delivered dose of irradiation. Increases in the delivered dose may increase the risk of photo reactivation and reduce the practicality of clinical implementation, while reduced dose delivery may reduce efficacy against ST5 and additional vegetative, spore-forming, and/or fungal pathogens [17,26]. 

## Conclusions

UV-C irradiation after bioburden removal via use of a wet microfiber cloth free of chemicals was a noninferior cleaning strategy to targeted disinfection cleaning with chemical wipes in the laboratory setting against the more pathogenic *S. aureus* ST 5. Future work should examine the efficacy of the approach against additional pathogens and evaluate clinical efficacy and effectiveness.
